# Gene array of VHL mutation and hypoxia shows novel hypoxia-induced genes and that cyclin D1 is a VHL target gene

**DOI:** 10.1038/sj.bjc.6601657

**Published:** 2004-02-24

**Authors:** C C Wykoff, C Sotiriou, M E Cockman, P J Ratcliffe, P Maxwell, E Liu, A L Harris

**Affiliations:** 1 Molecular Oncology Laboratories, John Radcliffe Hospital, Weatherall Institute of Molecular Medicine, Cancer Research UK, Oxford OX3 9DS, UK; 2 Division of Clinical Sciences, National Cancer Institute, USA; 3 Wellcome Trust Center for Human Genetics, Oxford OX3 7BN, UK

**Keywords:** VHL, hypoxia, cyclin D1, hypoxia inducible factor1, renal

## Abstract

Gene expression analysis was performed on a human renal cancer cell line (786-0) with mutated VHL gene and a transfectant with wild-type VHL to analyse genes regulated by VHL and to compare with the gene programme regulated by hypoxia. There was a highly significant concordance of the global gene response to hypoxia and genes suppressed by VHL. Cyclin D1 was the most highly inducible transcript and 14-3-3 epsilon was downregulated. There were some genes regulated by VHL but not hypoxia in the renal cell line, suggesting a VHL role independent of hypoxia. However in nonrenal cell lines they were hypoxia regulated. These included several new pathways regulated by hypoxia, including RNase 6PL, collagen type 1 alpha 1, integrin alpha 5, ferritin light polypeptide, JM4 protein, transgelin and L1 cell adhesion molecule. These were not found in a recent SAGE analysis of the same cell line. Hypoxia induced downregulation of Cyclin D1 in nonrenal cells via an HIF independent pathway. The selective regulation of Cyclin D1 by hypoxia in renal cells may therefore contribute to the tissue selectivity of VHL mutation.

Germline mutations in the von Hippel-Lindau (*VHL*) tumour suppressor gene are associated with a dominantly inherited renal cancer syndrome (Latif *et al*, 1993; Chen *et al*, 1995) and sporadic renal cancer. The *VHL* gene product pVHL is a critical component of a multiprotein ubiquitin ligase complex that targets the regulatory HIF-*α* subunits of hypoxia-inducible factor 1 (HIF-1) for oxygen-dependent proteolysis (Iwai *et al*, 1999; Maxwell *et al*, 1999; Cockman *et al*, 2000; Ivan *et al*, 2001). HIF-1 is expressed in response to hypoxia in most cell types and activates the transcription of genes involved in a variety of physiological and cellular processes including vascular endothelial growth factor (VEGF), glucose transport (glucose transporters), glycolysis (glycolytic enzymes), and cell survival (insulin-like growth factor 2) (Semenza, 1999). pVHL defective cells, both in cell culture and in the context of human tumours, constitutively overexpress HIF-1 target genes irrespective of their environmental oxygen concentration (Iliopoulos *et al*, 1996), due to the constitutive stabilisation of HIF-alpha subunits (Maxwell *et al*, 1999).

While a clear role of HIF-1 in producing vascularisation of tumours has emerged, the role of HIF activation in oncogenesis is still poorly defined. In particular, the mechanisms for tissue specificity of the effects of VHL mutation are unknown. Recently, we and others have identified a number of new pVHL target genes and demonstrated that many of these were hypoxia-responsive in wild-type pVHL cell lines (Koong *et al*, 2000; Wykoff *et al*, 2000; Lal *et al*, 2001). Whether there are other hypoxia non-VHL regulated pathways in renal cell lines is unknown.

Therefore in the current study, we have analysed a pair of renal cell carcinoma transfectants that are either defective or competent for pVHL to mRNA expression profiling. We have examined not only the pattern of gene expression affected by pVHL status but also that affected by hypoxia in both the absence and presence of a functional *VHL* gene product The analysis shows that there was a strong relation between genes regulated by hypoxia and those regulated by VHL. There were no genes regulated by hypoxia independently of VHL, but there were genes regulated by VHL that were not hypoxia regulated in the renal cell line used for the study. The unexpectedly high concordance prompted us to analyse hypoxia regulation of genes in the latter class in other cell types, and also genes from our previous study of VHL regulated genes in a different renal cell type (Wykoff *et al*, 2000), which were VHL but not hypoxia responsive. These genes were clearly regulated by hypoxia in other nonrenal cell types.

Cyclin D1 was identified as the most highly inducible gene in the array and in contrast to all other cell lines and studies reported, was upregulated by hypoxia not downregulated. Additionally, 14-3-3 epsilon was downregulated providing a combined modulation of G1 and G2 checkpoints. The tissue selectivity of VHL transformation in renal cancer may be related to the tissue-specific direction of regulation of this key checkpoint by hypoxia.

## MATERIALS AND METHODS

### Cell lines

786-0 cells expressing pVHL or empty vector were a gift from WG Kaelin. RCC lines were as described (Maxwell *et al*, 1999). RCC4/iVHL-HA (C1.2) and RCC4/iVA (VA1) are human renal cancer cell lines with mutant VHL, the former transfected with an HA tagged wild-type VHL, the latter the empty vector control. A549, EJ28, HBL-100, and ZR-75-1 lines were from ATCC. Embryonic stem cells with deleted HIF-1 alpha or HIF-2 alpha were generously provided by P Carmeliet. Cells were grown in DMEM (Sigma) supplemented with 10% foetal calf serum (Globepharm), L-glutamine (2 mM), penicillin (100 *μ*g ml^−1^), and streptomycin sulphate (100 U ml^−1^). Studies of inducible gene expression were performed on cells approaching confluence. Hypoxic conditions were generated in a Napco 7001 incubator (Precision Scientific) with 0.1% O_2_, 5% CO_2_, and balance N_2_, for 16 h.

### Preparation and hybridisation of fluorescent labelled cDNA

One round Eberwine's *et al* (1992) RNA amplification procedure with minor modifications was performed using polyA RNA from 786-0 RCC cancer cell line under different conditions. This methodology is similar to that employed by Affymetrix, Inc. for the production of probe in their chip microarray expression analysis.

The cDNA probes were prepared from antisense RNA. Briefly, we used 3 *μ*g of antisense RNA for Cy3 and Cy5 labelling. After probe purification the two separated probes were combined, mixed with hybridisation solution, denatured, and hybridised onto a 6000-feature cDNA microarray in a humidified chamber at 65°C for 16 h. The slides were then rinsed by submersion and agitation for 2 min in 2 × SSC with 0.1% SDS, followed by 1 × SCC, 0.2 × SCC, and 0.05 × SCC and then dried.

### Scanning and data processing

Following hybridisation, microarrays were scanned using a 10 *μ*m resolution GenePix 4000 scanner (Axon Instruments, Inc., Foster City, CA, USA) at variable PMT (photo-multiplier tube) voltage to obtain maximal signal intensity with <1% probe saturation. Resulting TIFF images for each fluorescent were analysed with GenePix software version 3.0 (Axon Instruments, Inc., Foster City, CA, USA). The data files generated by GenePix v3.0 were entered into a web-based database maintained by the Bioinformatics and Molecular Analysis Section of the CIT (Center for Information Technology), National Cancer Institute, Bethesda, MD, USA.

To study the gene expression profiles, an average linkage hierarchical cluster analysis utilising a correlation metric of similarity for clustering genes was performed as described by Eisen *et al* (1998). A metric multidimensional scaling for analysing and visualising the correlation among expression profiles of samples was also performed (Tenenbaum *et al*, 2000). To exclude labelling biases, antisense RNA-based targets from either cell line were labelled with the reciprocal fluorochrome in every other duplicate experiment. Differentially expressed genes were designated significant if they were reproducibly induced by >2-fold in three out of four experiments for each screening.

### Ribonuclease protection assay (RPA)

Total RNA was extracted by a modified acid/guanidinium thiocyanate/phenol/chloroform method (RNAzol B, Cinna/Biotec Laboratories), and dissolved in hybridisation buffer (80% formamide, 40 mM PIPES, 400 mM sodium chloride, and 1 mM EDTA, pH 8). For details of riboprobe templates employed to examine the expression of previously described VHL-responsive genes, see Wykoff *et al* (2000). Quantification of the protected species from 30 *μ*g was performed using a phosphoimager (Molecular Dynamics), and related to an internal control assay for the constitutively expressed U6 small nuclear RNA (*LC*), performed for each assay as described (Maxwell *et al*, 1999).

### Cell lysis and immunoblotting

Whole-cell protein extracts were prepared from tumours by section of frozen tissue and 30 s homogenisation in denaturing conditions as described (Wiesener *et al*, 1998). For Immunoblot analysis, aliquots were separated by SDS–polyacrylamide gel electrophoresis and transferred onto Immobilon-P membranes. Cyclin D1 was detected using the mouse monoclonal anti-human cyclin D1 mouse monoclonal Ab MCA1756 (Serotec, UK) (1 : 1000) at 4°C for 16 h. HRP-conjugated goat-anti-mouse immunoglobulin (DAKO) (1 : 1000) was applied for 1 h at room temperature (RT). ECL Plus (Amersham Pharmacia) was used for visualisation.

## RESULTS

### Comparison of VHL-responsive and hypoxia-responsive genes on gene array

Comparing VHL-deficient 786-0 cell line expressing vector backbone alone (786-0) or wild-type human VHL (786-0/VHL), 28 genes were repressed and 29 genes were induced by stable transfection of VHL (Table 1

**Table 1:** Candidate VHL-responsive genes in the 786-0

**IMAGE**			**Fold Reg**	**Fold Reg**
**ID**	**Abbreviation**	**Gene**	**VHL−/VHL+**	**H/N**
841641	CCND1	Cyclin D1	4.2	4.6
248828	ID3	Inhibitor of DNA binding 3	4	NS
756372	RARRES2	Retinoic acid receptor responder 2	4	NS
345032	RNASE6PL	Ribonuclease 6 precursor	3.8	3.3
1407750	IGFBP3	Insulin-like growth factor-binding protein-3	3.6	3.3
47359	EDN1	Endothelin 1	3.4	2
726086	TFPI2	Tissue factor pathway inhibitor 2	3.4	NS
346545	LAMB1	Laminin, beta 1	3.4	NS
110467	CAV2	Caveolin 2	3.3	NS
755506	ANXA4	Annexin A4	3.2	NS
156520	TNFAIP6	Tumour necrosis factor, alpha-induced protein 6=TSG-6	2.9	NS
272942	HP=DKFZp434K121	Hypothetical protein dkfzp434k121	2.9	3.7
40017	HCS	Cytochrome *c*	2.8	NS
163189	CD24	CD24	2.8	NS
272185	RPL27	Ribosomal protein L27	2.8	NS
325822	TGFA	Transforming growth factor, alpha	2.6	2.8
298268	BTG1	B-cell translocation gene 1	2.6	NS
279085	MYO9B	Myosin-IXB	2.6	NS
324873	ID2	Inhibitor of DNA binding 2	2.5	NS
230218	GCN5L1	General control of amino-acid synthesis (yeast) homolog-like 1	2.5	NS
83231	CYP2B	Cytochrome *P*450, subfamily IIB, polypeptide 6	2.5	NS
855624	ALDH1	Aldehyde dehydrogenase 1	2.5	NS
343990	LOC51226	COPZ2=nonclathrin coat protein zeta-COP	2.4	NS
725454	CKS2	Cyclin -dependent kinase regulatory subunit 2	2.3	NS
139681	NNAT	Neuronatin	2.3	NS
287327	IGF1	Insulin-like growth factor 1	2.3	NS
291057	CDKN2C	p18-(INK6)=cyclin-dependent kinase 6 inhibitor	2.2	NS
364934	DAPK1	Death-associated protein kinase 1	2	NS

142788	SERPINH2	CBP2=collagen binding protein-2=serine protease inhibitor, clade H, 2	0.16	NS
309482	MAPK12	Mitogen activated protein (MAP) kinase 12	0.18	NS
139009	FN1	Fibronectin	0.26	NS
504763	SDC4	Syndecan-4	0.27	0.49
82734	FACL2	Fatty-acid-coenzyme A ligase, long-chain 2	0.30	0.5
491066	ACVR1	Activin A receptor, type 1=serine/threonine protein kinase receptor R1	0.31	NS
266106	YWHAE	14-3-3 epsilon	0.32	NS
325160	NP25	Neuronal protein 25	0.33	NS
785415	LOC51763	SKIP=skeletal muscle and kidney enriched inositol phosphatase	0.34	NS
841059	CAPG	Capping protein (actin filament), gelsolin-like	0.36	NS
198904	APMCF1	APMCF1 protein	0.37	NS
489212	PDHA1	Pyruvate dehydrogenase alpha subunit	0.37	NS
825442	PTP4A2	Protein tyrosine phosphatase type IVA member 2	0.38	NS
769959	COL4A2	Collagen, type IV, alpha 2	0.38	NS
549383	PLAB	Prostate differentiation factor=MIC-1=macrophage inhibitory cytokine-1	0.38	0.43
384078	ATP6D	ATPase, H+ transporting, lysosomal (vacuolar proton pump), member D	0.38	NS
840844	HSPA5	GRP78=78 kDa glucose regulated protein precursor	0.40	NS
489453	SRM300	EST:KIAA0324	0.41	NS
588829	AARS	UG5 alanyl-tRNA synthetase	0.41	NS
193913	LYN	Tyrosine kinase	0.41	NS
136744	SPOP	Speckle-type, poxvirus and zinc-finger domain (POZ) containing, protein	0.42	NS
897177	PGAM1	Phosphoglycerate mutase 1 (brain)	0.42	NS
324437	GRO1	MGSA=melanoma growth stimulatory activity	0.42	NS
868304	ACTA2	Actin, alpha 2	0.43	NS
307553	KRAS2	v-Ki-ras2 Kirsten rat sarcoma 2 viral oncogene homolog	0.45	NS
810213	IL1R1	Interleukin-1 receptor, type I	0.46	0.45
742132	ISG15	Interferon-induced 17 kDa protein	0.47	NS
208161	GW128	GW128 protein	0.48	NS
325583	KIAA1538	EST:KIAA1538	0.48	NS

=, nomenclatures represent the same gene; Fold Reg VHL−/VHL+, fold-regulation of each gene by VHL-status as a ratio of expression in 786-0/expression in 786-0/VHL; Fold Reg H/N, fold-regulation of each gene by hypoxia in 786-0/VHL as a ratio of expression in hypoxia/expression in normoxia; NS, not significantly regulated.

). As anticipated, some of the VHL-repressible genes were previously identified VHL-targets, including endothelin 1 (EDN1) and transforming growth factor alpha (TGFA). In 786-0/VHL, 11 genes were induced and nine genes were repressed by hypoxia (Table 2

**Table 2:** Candidate hypoxia-responsive genes in 786-0/VHL

**IMAGE**			**Fold Reg**	**Fold Reg**
**ID**	**Abbreviation**	**Gene**	**H/N**	**VHL−/VHL+**
841641	CCND1	Cyclin D1	4.6	4.2
712341	RNASE6PL	Ribonuclease 6 precursor	3.3	3.8
272942	HP=DKFZp434K121	Hypothetical protein DKFZp434K121	3.7	2.9
1407750	IGFBP3	Insulin-like growth factor-binding protein-3	3.3	3.6
504527	DUSP1	CL100=MAP kinase phosphatase 1	3.2	NS
325822	TGFA	Transforming growth factor, alpha	2.8	2.6
51817	MFNG	Manic fringe (Drosophila) homolog	2.4	NS
460538	GSR	Glutathione reductase	2.1	NS
755578	SLC7A5	Solute carrier family 7 (cationic amino-acid transporter), member 5	2.1	NS
486436	UGP2	Uridine diphosphoglucose pyrophosphorylase	2.1	NS
47359	EDN1	Endothelin 1	2	3.4

549383	PLAB	Prostate differentiation factor=MIC-1=macrophage inhibitory cytokine-1	0.43	0.38
810213	IL1R1	Interleukin-1 receptor, type I	0.45	0.46
756092	HLA-DQA1	Major histocompatibility complex (MHC) class II, DQ alpha 1 chain	0.45	NS
307325	EDG1	Endothelial differentiation protein=putative G-protein-coupled receptor	0.46	NS
724397	CTSW	Lymphopain=C1 peptidase expressed in natural killer and cytotoxic T cells	0.49	NS
171693	D6S49E	LST1=leukocyte-specific transcript-1=interferon-gamma-inducible gene	0.49	NS
504763	SDC4	Syndecan-4	0.49	0.27
181998	NFATC4	Nuclear factor of activated T-cells, cytoplasmic, calcineurin-dependent 4	0.50	NS
82734	FACL2	Fatty-acid-Coenzyme A ligase, long-chain 2	0.50	0.3

=, nomenclatures represent the same gene; Fold Reg VHL−/VHL+, fold-regulation of each gene by VHL-status as a ratio of expression in 786-0/expression in 786-0/VHL; Fold Reg H/N, fold-regulation of each gene by hypoxia in 7860/VHL as a ratio of expression in hypoxia/expression in normoxia; NS, not significantly regulated.

).

**Figure 1:**
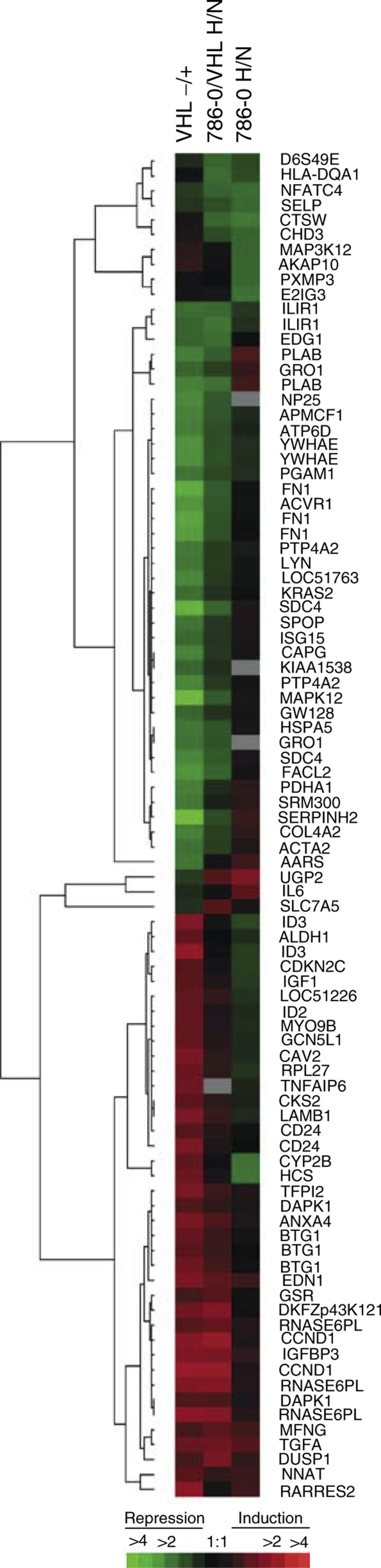
Hierarchical cluster analysis of differentially expressed genes. Each row represents a single gene (identified by its abbreviation at the right. See Table 1, 2, or 3 for corresponding gene name). Each column represents the average of the four replicates for each experiment. VHL−/+, comparison of gene expression in 768-0 (−) *vs* 786-0/VHL (+) in normoxia; 786-0/VHL H/N, comparison of gene expression in hypoxia (H) *vs* normoxia (N) in 786-0/VHL; 786-0 H/N, comparison of gene expression in hypoxia (H) *vs* normoxia (N) in 786-0. Significantly regulated genes are represented by a block of a particular colour, which was determined by its sign and magnitude of regulation by the given stimulus; red blocks indicate overexpressed genes while green blocks indicate underexpressed genes. Black bars indicate genes with approximately equivalent expression levels and grey bars indicate missing data. Colour code at bottom correlates colour intensity with fold-regulation. Dendrogram at the left of the figure illustrates the relationship between the observed patterns of gene regulation, where the shorter the branch length between two gene, the more similar their pattern of regulation across the three comparisons.

A striking concordance in the pattern of gene regulation across the entire screen was observed when the VHL-responsive genes were compared to the hypoxia-responsive genes in 786-0/VHL (Figure 1

). VHL-repressible genes (represented with reds of various intensities in the left column) were typically hypoxia-inducible in 786-0/VHL (red in middle column). A similar response was seen with VHL-inducible genes, which were hypoxia repressible (represented with greens of various intensities in the left column).

In comparison to the changes in gene expression induced by changes in VHL-status and hypoxia in 786-0/VHL, strikingly few changes in gene expression were observed in 786-0 in response to hypoxia. Three genes were significantly induced by hypoxia in 786-0, two of which, TGFA and uridine diphosphoglucose pyrophosphorylase (UGP2), were also identified as hypoxia-inducible in 786-0/VHL. Array screening identified 10 genes that were repressed by hypoxia in 786-0 (Table 3

**Table 3:** Candidate hypoxia-responsive genes in 786-0

**IMAGE**			**Fold Reg**
**ID**	**Abbreviation**	**Gene**	**H/N**
486436	UGP2	Uridine diphosphoglucose pyrophosphorylase	3.4
310406	IL6	Interleukin-6	2.3
325822	TGFA	Transforming growth factor, alpha	2.2

724397	CTSW	Lymphopain=C1 peptidase expressed in natural killer and cytotoxic T cells	0.40
40017	HCS	Cytochrome *c*	0.42
83231	CYP2B	Cytochrome *P*450, subfamily IIB, polypeptide 6	0.43
262053	E2IG3	Putative nucleotide binding protein, oestradiol-induced	0.43
181998	NFATC4	Nuclear factor of activated T-cells, cytoplasmic, calcineurin-dependent 4	0.46
182264	SELP	P-selectin=CD62=GMP140=granulocyte membrane protein 140	0.48
788518	PXMP3	Peroxisomal membrane protein 3 (35 kDa, Zellweger syndrome)	0.48
43630	AKAP10	Kinase A anchor protein 10	0.48
1071934	MAP3K12	Mitogen-activated protein kinase kinase kinase 12	0.49
359021	CHD3	Zinc-finger helicase (hZFH)	0.50

=, nomenclatures represent the same gene. Fold Reg H/N, fold-regulation of each gene by hypoxia in 786-0 as a ratio of expression in hypoxia/expression in normoxia.

)

### RNase protection analysis of candidate VHL-responsive and hypoxia-responsive genes

**Figure 2:**
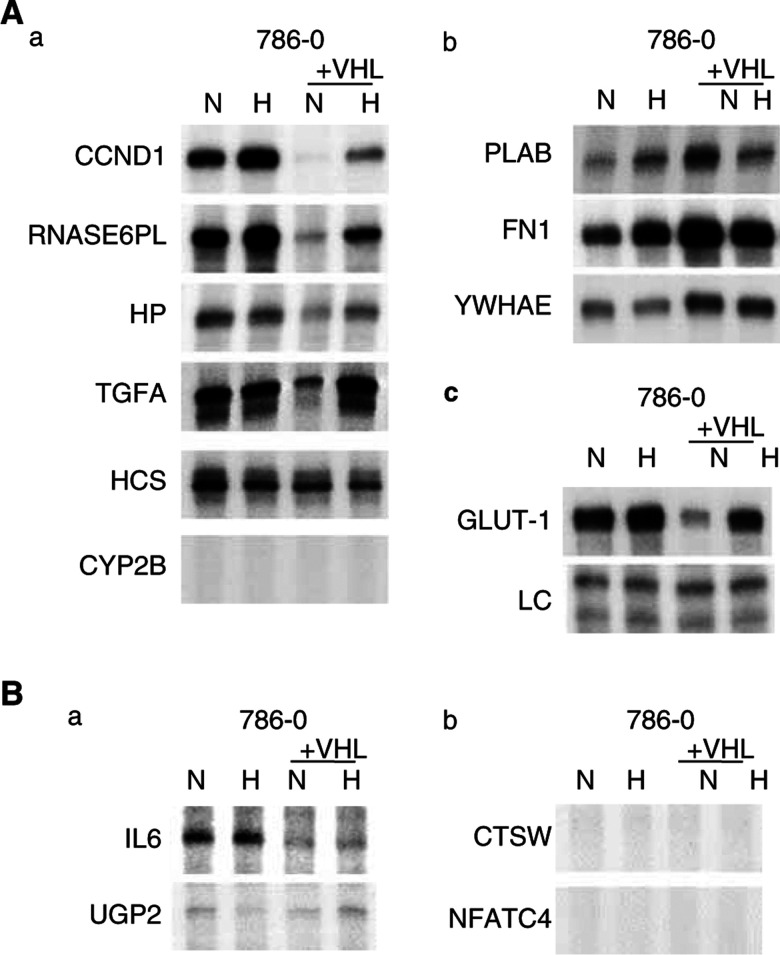
RPAs of array screening predicted VHL and hypoxia-responsive target genes. Cells were exposed to either normoxia (N; 20% O_2_) or hypoxia (H; 0.1% O_2_) for 16 h. Analysis of expression in 786-0 and the corresponding wt VHL stable tranfectant (+VHL) by RPA. See Table 1, 2 and 3 for corresponding gene names and a summary of the illustrated RPAs. (**A**) Genes predicted to be either downregulated (a) or upregulated (b) by wt VHL. (c) Expression of control genes: GLUT-1, a known hypoxia-inducible VHL target; LC, internal control assay (constitutively expressed U6 small nuclear RNA). All samples and assays (in **A** and **B**) were controlled in both ways. (**B**) Genes predicted to be either upregulated (**a**) or downregulated (**b**) by hypoxia in 786-0.

RNase protection assays (RPA) were used to validate the findings. Nine of the identified VHL-responsive genes predicted by array screening to be either downregulated (cyclin D1 (CCND1), ribonuclease 6 precursor (RNASE6PL), hypothetical protein DKFZp434K1210 (HP), TGFA, cytochrome *c* (HCS), and cytochrome *P*450 subfamily IIB polypeptide 6 (CYP2B)), or upregulated (prostate differentiation factor (PLAB), fibronectin (FN1), and 14-3-3 epsilon (YWHAE)) by reintroduction of VHL into 786-0 were examined by RPA (Figure 2A(a) and (b)

respectively, comparing the paired cell lines in normoxia). Seven of these genes were clearly confirmed to be regulated as predicted by array screening, while one did not show regulation (HCS) and one was not expressed at detectable levels by RPA (CYP2B).

Five of these predicted VHL-responsive genes were also predicted by the array analysis to be regulated by hypoxia in 786-0/VHL. As illustrated in Figure 2A, each of these genes were confirmed to be either upregulated (CCND1, RNASE6PL, HP, TGFA) or downregulated (PLAB) by hypoxia in 786-0/VHL.

Therefore, each analysed gene predicted by array screening to be regulated both by VHL-status and by hypoxia in 786-0/VHL was confirmed by RPA, that is, the genes responsive to hypoxia were also regulated by VHL-status in 786-0/VHL.

### VHL independent hypoxia response

To examine for the presence of VHL-independent hypoxia-mediated changes in gene expression, seven of the hypoxia-responsive genes predicted by array screening in 786-0 were examined by RPA as being either upregulated (TGFA, IL6, and UGP2) or down-regulated (HCS, CTSW, CYP2B, and NFATC4) by hypoxia (Figure 2B(a) and (b) respectively). This included all of the genes predicted to be significantly hypoxia-inducible in 786-0, and four of the top five differentially expressed genes predicted to be significantly downregulated by hypoxia in 786-0. While one of these genes, HCS, was slightly regulated as predicted by array screening, TGFA and IL6 were expressed but unregulated, UGP2 was too lowly expressed to make a definitive conclusion as to regulation, and CTSW, CYP2B, and NFATC4 were not expressed at detectable levels by RPA.

Therefore, while array screening for genes responsive to VHL-status and hypoxia in 786-0/VHL was generally reliable, the majority of genes predicted to be hypoxia-responsive in 786-0 were unable to be confirmed by RPA.

### Further characterisation of cyclin D1 RNA and protein expression in renal carcinoma derived cell lines

**Figure 3:**
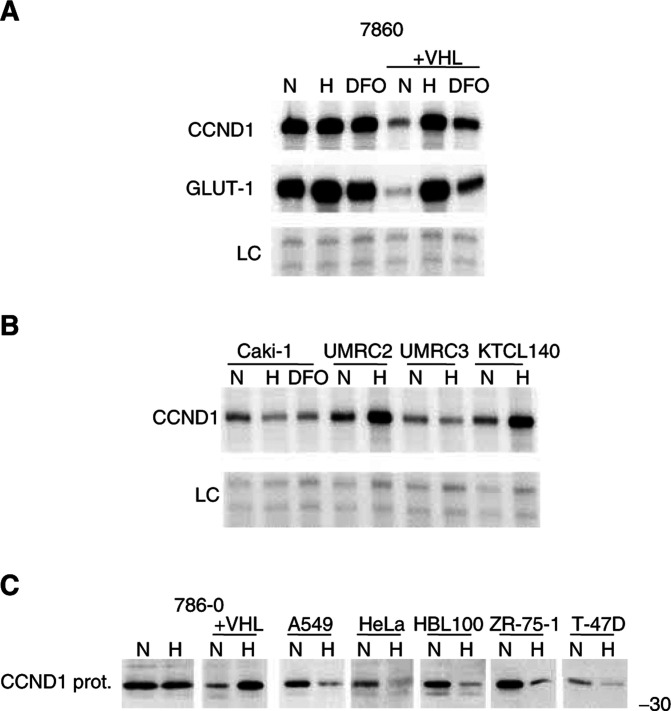
Further characterisation of the cyclin D1 response to hypoxia. Cells were exposed to either normoxia (N; 20% O_2_), hypoxia (H; 0.1% O_2_), or desferrioxamine (DFO; 100*μ*M) for 16 h. (**A**) RPAs of cyclin D1 (CCND1) and GLUT-1 in either 786-0 or 786-0/VHL exposed to either N, H, or DFO. (**B**) RPAs of CCND1 in Caki-1 (pVHL functionally wt), UMRC2, UMRC3, and KTCL140 (pVHL functionally deficient). LC, internal control assay (constitutively expressed U6 small nuclear RNA). (**C**) Western blots of whole-cell extracts using anti-human CCND1 monoclonal Ab MCA1756; CCND1 protein expression in kidney (786-0, 786-0/VHL), lung (A549), cervical (HeLa), and breast (HBL100, ZR-75-1, T-47D) derived cell lines. Numbers to the right of protein gels indicate approximate molecular weights (kDa) as determined by protein standards run on each gel.

The expression of CCND1 was examined in three additional renal carcinoma cell lines, and its regulation by the iron chelator desferrioxamine (DFO) (Figure 3A

). CCND1 RNA was not affected by either hypoxia or DFO in the functionally wt VHL cell line, Caki-1. It was not modified by hypoxia in the VHL-defective cell lines, UMRC2, UMRC3, and KTCL140 (Figure 3B). CCND1 protein was found to be regulated by VHL-status and hypoxia in the 786-0 background similarly to the regulation of its RNA (Figure 3C).

### CCND1 regulation by an inducible VHL in renal cancer cell line RCC4

**Figure 4:**
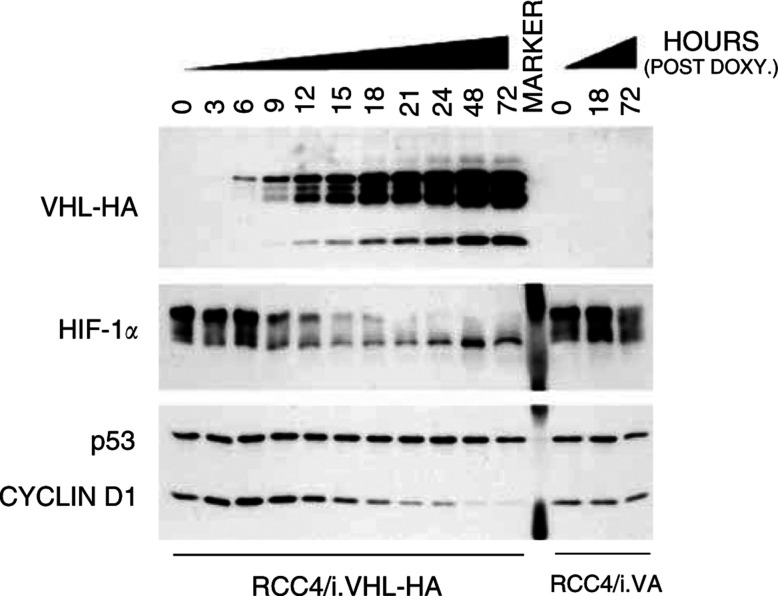
Time course of DOX-inducible pVHL-HA protein expression and effect upon downstream targets. Parallel cultures of RCC4/iVHL-HA (C1.2) and RCC4/iVA (VA1) were grown in the presence of DOX (0.5 *μ*g ml^−1^) for up to 72 h. Whole-cell protein extracts were collected subconfluent populations of C1.2 and VA1 cells at 3-h intervals for the first 24 h, and daily thereafter. Whole-cell extracts (20 *μ*g) were resolved by SDS–PAGE: VHL-HA (13.5%); HIF-1*α* (6%); and cyclin D1/p53 (10%) and immunoblotted with: anti-HA (rat mono); anti-HIF-1*α* (mAb clone 54); anti-cyclin D1 (pAb MCA1756); and anti-p53 (mAb DO-7). Equivalent loading was confirmed by Coomassie staining membrane after immunoblotting.

To better characterise CCND1 regulation, the effect of regulated pVHL expression upon CCND1 protein expression was examined in RCC4/iVHL-HA and RCC4/iVA cells the former comprising an inducible wild-type VHL in a VHL minus background, the latter the control. A 72 h time course of DOX treatment was employed to assess the relationship between pVHL induction and CCND1 expression (Figure 4

). There was a time-dependent increase in pVHL-HA protein expression. The kinetics of pVHL-HA expression was rapid and over a large inducible range. HIF-1*α* protein expression highlighted an inverse relationship with pVHL expression (Figure 4, middle panel). In contrast to p53 expression, which was not affected by pVHL status, the expression of the CCND1 gene product was almost completely suppressed following pVHL-HA induction. Eliminating the possibility of nonspecific DOX-associated side effects, CCND1 expression was not modulated in control cells treated in parallel.

### Analysis of cyclin D1 mRNA and protein expression in nonrenal cell lines

CCND1 protein has been reported to be downregulated by hypoxia in at least two cell lines: the pheochromocytoma derived PC12 cell line (Conrad *et al*, 1999) and the ovarian carcinoma SKA cell line (Krtolica *et al*, 1999). To assess the tissue specificity of responses cell lines derived from the lung (A549), bladder (EJ-28), breast (HBL100), or cervical (HeLa) tissue were used to analyse CCND1 regulation. CCND1 RNA was constitutively expressed and unresponsive to hypoxia in two cell lines (A549 and EJ-28), had very low level expression in HBL100 cells, and downregulated by hypoxia in HeLa cells (data not shown).

CCND1 protein was downregulated by hypoxia in all the nonrenal cell lines examined including cells derived from lung (A549), cervical (HeLa), or breast (HBL100, ZR-75-1,T-47D) cancers (Figure 3C).

### Expression of cyclin D1 in HIFalpha mutant cells

**Figure 5:**
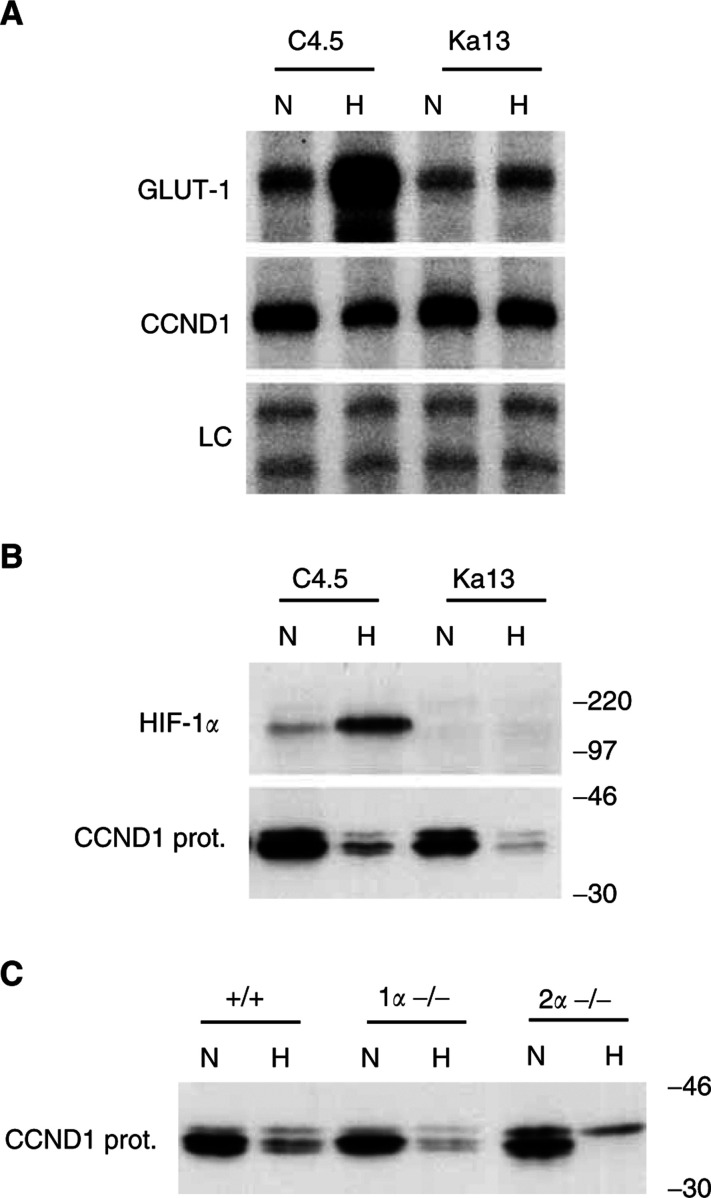
Response of cyclin D1 to hypoxia is HIF-1*α* and HIF-2*α* independent in non-RCC cells. Cells were exposed to either normoxia (N; 20% O_2_) or hypoxia (H in non-RCC cells; 0.1% O_2_) for 16 h unless otherwise stated. (**A** and **B**) Examination of wild-type CHO cells (C4.5) and HIF-1*α* deficient CHO cells (Ka13). (**A**) RPAs of GLUT-1 and cyclin D1 (CCND1). LC, internal control assay (constitutively expressed U6 small nuclear RNA). (**B** and **C**) Western blots of whole-cell extracts using either anti-human HIF-1*α* mouse monoclonal Ab NB 100-105 or anti-human CCND1 monoclonal Ab MCA1756. (**B**) HIF-1*α* and CCND1 protein expression. (**C**) Induction of CCND1 protein by hypoxia (1% O_2_, 16 h) in mouse wt (+/+), HIF-1*α* knockout (1*α*−/−) and HIF-2*α* knockout (2*α*−/−) embryonic stem cells. Numbers to the right of protein gels indicate approximate molecular weights (kDa) as determined by protein standards run on each gel.

CCND1 response to hypoxia was examined in a wild-type Chinese hamster ovary cell line (C4.5) and a mutant derivative that is functionally defective in HIF-1 alpha (Ka13) (Wood *et al*, 1998). While CCND1 RNA was slightly downregulated by hypoxia in both C4.5 and Ka13 (Figure 5A

), its protein showed a more significant downregulation by hypoxia, which was HIF alpha-independent (Figure 5B). CCND1 protein response to hypoxia was examined in wt, HIF-1 alpha knockout (^−/−^), and HIF-2 alpha ^−/−^ ES. As illustrated in Figure 5C, CCND1 protein was downregulated by hypoxia independent of HIF-1 alpha or HIF-2 alpha in this background.

### Expression of cyclin D1 in human renal cell carcinomas

CCND1 protein expression was examined in protein extracts from a panel of renal cell carcinomas (RCCs) (T) of either the clear cell (CC-RCC) or papillary type and compared to CCND1 protein expression in adjacent macroscopically normal tissue (N) from the same patient. In contrast to the four papillary tumours, nine of 10 CC-RCCs expressed markedly elevated levels of CCND1 protein (data not shown).

### Regulation of other genes by VHL but not hypoxia

There were more genes regulated by VHL re-expression than regulated by hypoxia in the array. As the experiment with inducible VHL showed a wide range of expression with time and a similar reciprocal regulation of CCND1, it is possible that a transfectant with a set high level of VHL could be sufficient to continue to repress some genes in hypoxia. The nonrenal cell lines were therefore analysed for hypoxic regulation of genes that showed weak regulation in the transfectant. FN1 was hypoxia-inducible in both (A549, HBL100) nonrenal cell lines in which its RNA was detected.

**Figure 6:**
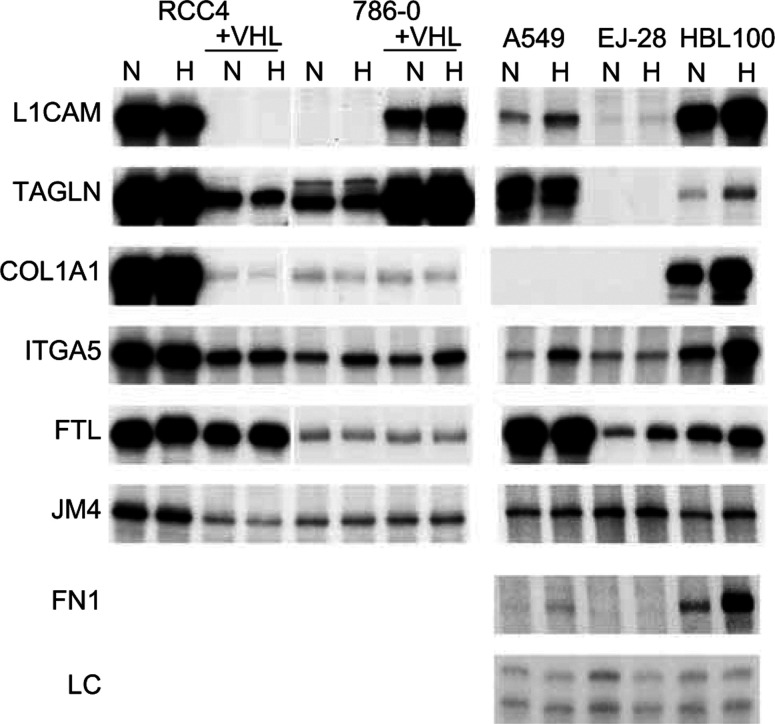
Comparison of the hypoxic regulation of genes modulated by VHL in renal cell lines with their regulation in nonrenal cell lines. COL1A1 collagen type 1, alpha 1, ITGA5 integrin, alpha 5,— FTL ferritin, light polypeptide, JM4 JM4 protein, TAGLN transgelin, L1CAM L1 cell adhesion molecule; FN1, fibronectin; LC, loading control.

Of importance in interpreting these results, a similar phenomenon was observed in our previous study of VHL-responsive genes in the RCC4 background. In view of the results here we reanalysed six of these genes (collagen type 1alpha 1, integrin alpha 5, ferritin light polypeptide, JM4 protein, transgelin, L1 cell adhesion molecule) in the nonrenal cell lines here and they were all hypoxia-responsive in at least one of them (Figure 6

).

However, another observation in the renal cell lines was that VHL expression had opposite effects in the 786-0 and the RCC4 on certain genes. Restoration of VHL suppressed LICAM and transgelin in the RCC4 cells but induced expression in the 786-0 cells. Thus hypoxia regulation in opposite directions can occur in different cell lines, again reflecting cell type specific operation of hypoxia pathways.

## DISCUSSION

This study has provided insight into the role of VHL in the global response to hypoxia. Specifically, at least in the 786-0 background, VHL plays a central and dominant role in eliciting the changes in gene expression generated by hypoxia. In contrast, there was no further consistent gene regulation in response to hypoxia in the absence of a functional VHL gene product.

These results should be compared to the recent SAGE analysis of the same renal cell lines (Jiang *et al*, 2003). These authors found 38 genes induced by hypoxia in the 786-0/VHL cell in common with those downregulated by re-expression of VHL in 786-0 cells. It is of interest that not one of the genes we found to be regulated in common by the gene array and validated by RNase protection is in that list and our list contains genes previously validated in other studies on renal cancer. This emphasises the importance of different methodologies in analysing gene expression. Some of the differences could be due to different cut points of analysis as well as inherent methodology differences, for example in sequencing and hybridisation.

A novel observation was that a large proportion of genes from our array experiments were significantly regulated by VHL-status but insignificantly regulated by hypoxia in VHL transfectants, while hypoxia-responsive in nonrenal cell lines. One potential factor contributing to this observation is that stable over-expression of exogenous pVHL to a level in excess to that which had previously been present in the precursor cells of the cancer may have an inhibitory effect on some hypoxia-mediated changes in gene expression. Another is that these genes belong to hypoxia pathways that have become modified during progressive development of renal cancer and are no longer responsive. An interesting possibility is that they represent a VHL pathway independent of HIF. Jiang *et al* (2003) found many more genes by SAGE that were VHL regulated but not hypoxia responsive but they did not test whether they were hypoxia inducible in other cell types.

Other genes we identified as hypoxia regulated in nonrenal cell lines have roles that could contribute to the malignant phenotype, including cell adhesion and VEGF presentation, L1CAM (Castellani *et al*, 2002), iron metabolism or gelling of the actin cytoskeleton, transgelin.

The genes identified from the current analyses broaden the spectrum of VHL and hypoxia-responsive target genes to include many that have functions of interest to cancer biology. Tumour growth factor alpha, found to be upregulated by VHL mutation and hypoxia in this screen, has recently been shown to have a key role in growth of renal cancers. 14-3-3-epsilon, a cell cycle inhibitor that complexes with cdc2 kinase (van Hemert *et al*, 2001) was downregulated by VHL mutation and provides a suppression of a second cell cycle checkpoint that would synergise with cyclin D1 changes.

The gene most upregulated by hypoxia and VHL mutation was cyclin D1. Over the past decade, the expression of CCND1 has emerged as being clearly disregulated in a variety of human neoplasms and having a major role in tumorigenesis. Recently Klausner's group specifically analysed the control of the cell cycle in the same cell line and found hypoxia upregulation of CCND1 and maintenance of CCND1 at confluence, which was not related to protein stabilisation. Similarly to their study, this only occurred in cell lines that had lost VHL function and been transfected with VHL (Bindra *et al*, 2002). In contrast, Maher's group used an array of 558 genes and found CCND1 upregulated in a VHL mutant renal cancer cell line (RCC4), but it was not regulated by hypoxia (Zatyka *et al*, 2002). This may be due to the problem discussed above where transfection may not restore hypoxia inducibilty of genes in such cells. Our results in that cell line with an inducible VHL clearly show that VHL upregulation produces suppression of CCND1, emphasing the importance of trying to titrate the level of a suppressor gene to determine the effects.

CCND1 protein was downregulated by hypoxia in multiple nonrenal cell lines as previously reported (Conrad *et al*, 1999; Krtolica *et al*, 1999), and we show here that this is by an HIF-alpha independent mechanism as shown in two different mutant cell types for HIF1-alpha and one for HIF2-alpha. The mechanism varied in the nonrenal cell lines, for example, in A549 cells the RNA was not hypoxia-responsive in while its protein was down-regulated, whereas in HeLa cells both were downregulated. Taken together, these results indicate that the controls responsible for modulating the CCND1 response to hypoxia are complex and cell type specific. Nevertheless, the renal cell lines stand out as consistently maintaining CCND1 expression under hypoxia in contrast to all other types examined here and reported in the literature. Our findings are in agreement with clinical data that approximately 75% of RCCs expressed a higher level of CCND1 protein than the normal kidney cortex (Lin *et al*, 1998; Aaltomaa *et al*, 1999; Hedberg *et al*, 1999; Stassar *et al*, 2001).

The renal specificity of transformation by VHL could be partly determined by the signalling pathway we describe here. The mechanism of CCND1 regulation will require further analysis, but a possibility is indirect regulation via TGF alpha or other growth factors regulated by HIF. Differential tissue specific regulation of genes by hypoxia provides a putative reason, for the remarkable specificity of VHL to produce invasive renal tumours. Our work shows three contributing factors – the upregulation of CCND1 in the presence of VHL mutation, downregulation of 14-3-3 epsilon, and lack of an HIF independent hypoxia response that could mediate the suppression of CCND1, as in other cell types. The global gene array has shown that essentially all hypoxia-regulated genes are regulated by VHL in renal cancer contributing to an understanding of tissue selectivity of transformation.
